# Coaching to vision versus coaching to improvement needs: a preliminary investigation on the differential impacts of fostering positive and negative emotion during real time executive coaching sessions

**DOI:** 10.3389/fpsyg.2015.00455

**Published:** 2015-04-24

**Authors:** Anita R. Howard

**Affiliations:** Department of Organizational Behavior, Weatherhead School of Management, Case Western Reserve UniversityCleveland, OH, USA

## Abstract

Drawing on intentional change theory (ICT; Boyatzis, 2006), this study examined the differential impact of inducing coaching recipients’ vision/positive emotion versus improvement needs/negative emotion during real time executive coaching sessions. A core aim of the study was to empirically test two central ICT propositions on the effects of using the coached person’s Positive Emotional Attractor (vision/PEA) versus Negative Emotional Attractor (improvement needs/NEA) as the anchoring framework of a onetime, one-on-one coaching session on appraisal of 360° feedback and discussion of possible change goals. Eighteen coaching recipients were randomly assigned to two coaching conditions, the coaching to vision/PEA condition and the coaching to improvement needs/NEA condition. Two main hypotheses were tested. Hypothesis_1_ predicted that participants in the vision/PEA condition would show higher levels of expressed positive emotion during appraisal of 360° feedback results and discussion of change goals than recipients in the improvement needs/NEA condition. Hypothesis_2_ predicted that vision/PEA participants would show lower levels of stress immediately after the coaching session than improvement needs/NEA participants. Findings showed that coaching to vision/the PEA fostered significantly lower levels of expressed negative emotion and anger during appraisal of 360° feedback results as compared to coaching to improvements needs/the NEA. Vision-focused coaching also fostered significantly greater exploration of personal passions and future desires, and more positive engagement during 360° feedback appraisal. No significant differences between the two conditions were found in emotional processing during discussion of change goals or levels of stress immediately after the coaching session. Current findings suggest that vision/PEA arousal versus improvement needs/NEA arousal impact the coaching process in quite different ways; that the coach’s initial framing of the session predominantly in the PEA (or, alternatively, predominantly in the NEA) fosters emotional processing that is driven by this initial framing; and that both the PEA (and associated positive emotions) and NEA (and associated negative emotions) play an important and recurrent role in shaping the change process. Further study on these outcomes will enable researchers to shed more light on the differential impact of the PEA versus NEA on intentional change, and how to leverage the benefits of both emotional attractors. Findings also suggest that coaches can benefit from better understanding the importance of tapping intrinsic motivation and personal passions through coaching to vision/the PEA. Coaches additionally may benefit from better understanding how to leverage the long-term advantages, and restorative benefits, of positive emotions during coaching engagements. The findings also highlight coaches’ need to appreciate the impact of timing effects on coaching intentional change, and how coaches can play a critical role in calibrating the pace and focus of work on intentional change. Early arousal of the coachee’s PEA, accompanied by recurrent PEA–NEA induction, may help coachees be/become more creative, optimistic, and resilient during a given change process. Overall, primary focus on vision/PEA and secondary focus on improvement needs/NEA may better equip coaches and coaching recipients to work together on building robust learning, development, and change. Keywords-133pt executive coaching, vision, improvement needs, positive emotion, negative emotion, emotional appraisal, intentional change, positive psychology

## Introduction

Executive coaching is a far-reaching practice to enhance the performance of 21st century professionals facing constant workplace change, challenge, and stress. Coaching is generally understood to involve practical, goal-focused forms of one-on-one learning and behavioral change (Peterson and Hicks, 1996; Hall et al., 1999). Promoting learning and behavior change in coaching contexts involves work on intentional change.

Intentional change is deliberate, altering, demanding. It results from the conscious effort to establish new behaviors that are different from what they currently are or appear to be (Ford and Ford, 1994). A key challenge for coaches is finding ways to support coachees for practicing new behaviors and/or building new habits and competencies. Although problem-focused coaching is an accepted approach to intentional change, positive psychology theory and research support the idea that vision-focused coaching helps coachees be more energized and resilient during work on desired change as compared to problem focused coaching. As Kauffman (2006) argues, “an explicitly positive psychology framework suggests that a language of strength and vision rather than weakness and pain be the firm foundation on which the coaching work rests” (Kauffman, 2006, p. 220).

The proposed shift from problem-focused coaching to vision-focused coaching does not imply that the “Pollyanna Principle” (i.e., excessive optimism) should drive the coaching agenda. Instead, coaches are encouraged to help their clients move more deftly between attention to vision and values, and attention to problems and improvement needs. Intentional change theory (ICT) proposes that executive coaches who anchor a coaching process in the coaching recipient’s vision *(PEA framing/early induction of positive affect)* trigger positive cognitive emotional processing associated with the broaden and build effect (Fredrickson, 1998, 2000a, 2001, 2003, 2013; Fredrickson and Joiner, 2002), i.e., ways in which positive emotions broaden and build thought-action repertories and attentional focus; speed recovery from negative emotional experiences and crises; optimize emotional well-being, physical health, and resilience; and undo the damaging effects of negative emotion (Fredrickson, 2000b; Fredrickson et al., 2000; Gottman et al., 2002; Tugade et al., 2004; Isen and Reeve, 2005; Tugade and Fredrickson, 2007).

**FIGURE 1:**
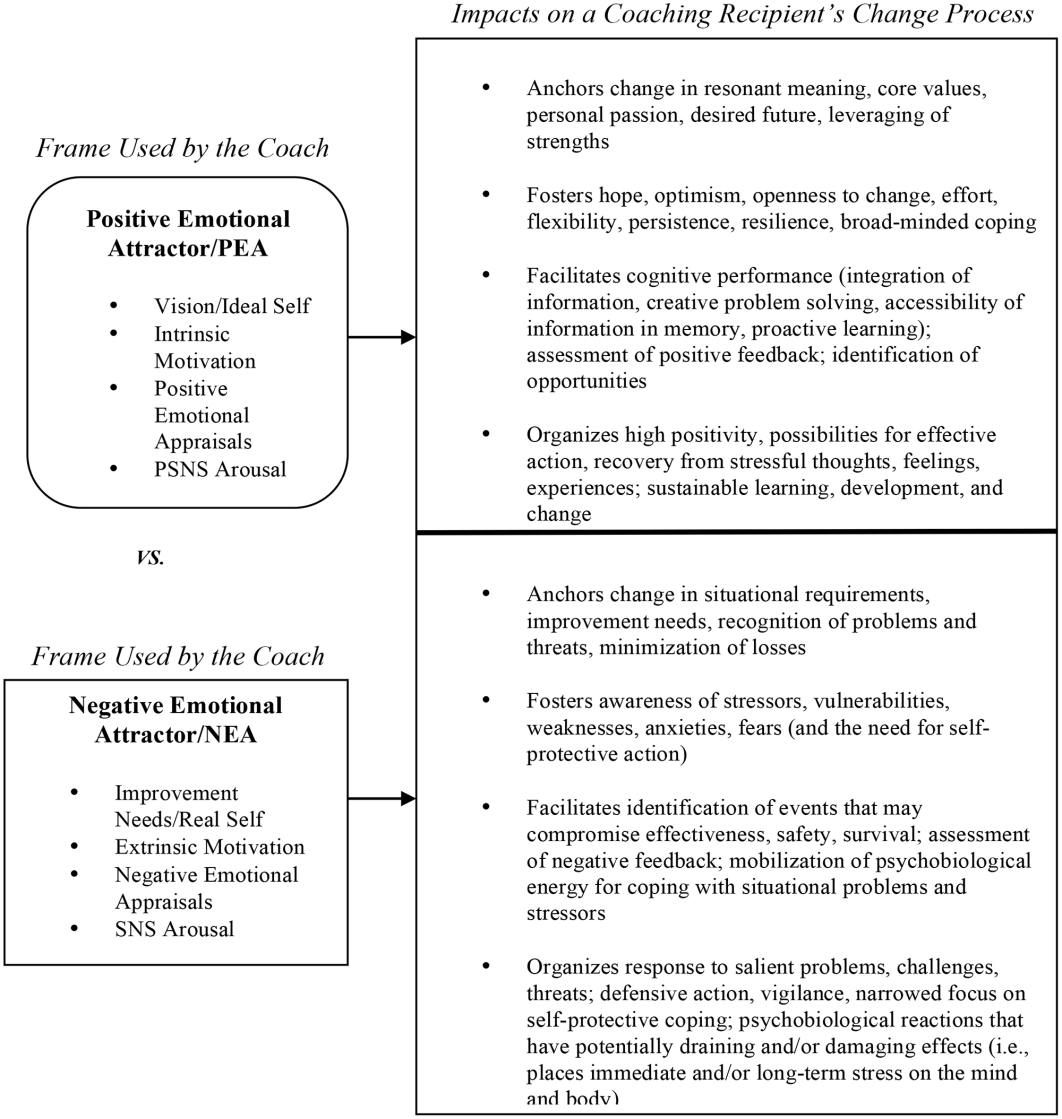
**Differential impacts of PEA framing versus NEA framing on a coaching recipient’s change process**.

Conversely, executive coaches who anchor a coaching process in the coaching recipient’s improvement needs *(NEA framing/early induction of negative affect)* trigger negative cognitive affective processing associated with adaptive response to extrinsic requirements and/or threats, i.e., ways in which negative emotions assist rapid recognition of problems, appraisal of negative feedback, evaluation of weaknesses, surfacing of fears and anxieties, and mobilization of psychophysiological energy for coping with situational concerns (French, 2001; Sanford and Rowatt, 2004; Parrott, 2014). **Figure 1** presents the proposed differential impacts of PEA framing versus NEA framing.

This paper empirically tests these ICT propositions and offers suggestive empirical support for the advantages of coaching to vision [i.e., coaching to the Positive Emotional Attractor (PEA)] versus coaching to problems and improvement needs [i.e., coaching to the Negative Emotional Attractor (NEA)]. Specifically, the study tested two main hypotheses on the real-time effects of using the coached person’s vision/PEA versus improvement needs/NEA as the anchoring framework of a one time, one-on-one coaching session on appraisal of 360° feedback and discussion of possible change goals:

*Hypothesis_1:_ Level of Positive Emotion During Appraisal of 360-degree Feedback Results and Discussion of Change Goals.* Participants coached using their vision/PEA as the primary focus on the coaching session will show higher levels of expressed positive emotion during appraisal of 360° feedback results and discussion of possible change goals than participants coached using their improvement goals/NEA as the primary focus.
*Hypothesis_2:_ Level of Stress Immediately After the Coaching Session.* Participants coached using their vision/PEA as the primary focus of the coaching session will exhibit lower levels of stress immediately after the coaching session than participants coached using their improvement needs/NEA as the anchoring framework.

## Background

### Role of the Positive Emotional Attractor (PEA) and Negative Emotional Attractor (NEA) in Intentional Change

Intentional change theory (Boyatzis, 2006) offers an evidence-based perspective on the role of positive and negative emotion in desired, sustained change, i.e., that positive emotions (aroused by the PEA) trigger constructive cognitive and physiological responses that enhance motivation, effort, optimism, flexibility, creative thinking, resilience, and other adaptive behaviors. Negative emotions (aroused by the NEA) trigger a different process by calling attention to current social and environmental challenges and stressors that may compromise one’s effectiveness. While both positive and negative emotions play an important role in intentional change, it is critically important to leverage the beneficial effects of positive affect (aroused by the PEA) throughout the change process.

The PEA is defined as the personal values, hopes, dreams, possibilities, strengths, optimism, and self-directed learning goals that make up the Ideal Self, i.e., our vision of what we most aspire to be and become (Boyatzis, 2006; Boyatzis and Akrivou, 2006). The organizing power of vision/the PEA stems from positive emotions (and emotional appraisals) aroused by affirming thoughts, feelings, memories, meaning, and self-worth that constitute the Ideal Self — and by arousal of the parasympathetic nervous system (PSNS) and neural circuits predominantly in the left prefrontal cortex. When intentional change is initiated by connecting to vision/the PEA, change becomes grounded in intrinsic motivation, personal passion, resonant meaning, belief in possibility and the psychophysiological benefits of PSNS arousal and neurogenesis.

The NEA is defined as the present reality, requirements, problems, shortfalls, fears, pessimism, and improvement goals that constitute the Real Self (Boyatzis, 2006; Taylor, 2006), i.e., our conception of what we actually are in everyday life. The organizing power of the NEA stems from negative emotions (and emotional appraisals) associated with and aroused by dissonant thoughts, feelings, memories, meaning and concerns about self-efficacy that comprise the Real Self — and by NEA arousal of the sympathetic nervous system (SNS) and neural circuits predominantly in the right prefrontal cortex.

During intentional change, negative emotions aroused by the NEA help the individual remain cognizant about salient environmental requirements and personal improvements that must be made. Negative emotions also support analysis of what needs to be done first, (priority setting), what stands in the way (obstacles, barriers), what resources are lacking, and what is not presently working (Diamond and Aspinwall, 2003). This information is central to the development of change goals and helps the individual to outline realistic approaches to behavior change.

The drawback of negative emotional arousal is that it keeps the person more narrowly focused on the challenges of present reality and introduces psychophysiological reactions that trigger self-protective cognitive and physiological response, but at the cost of “directing blood to large muscle groups, closing down non-essential neural circuits, suspending the immune system, and producing cortisol” (cortisol’s upside is that it catalyzes defensive response, but the downside is that cortisol inhibits neurogenesis and “overexcites older neurons, rendering them useless”; Boyatzis, 2006, p. 25).

Because episodes of negative emotional arousal tax the mind and body, and because intentional change is characterized by recurrent arousal of negative emotions, it is important to leverage the restorative effects of positive emotions throughout the change process. Failure to leverage the restorative effects of positive emotion compromises recovery from negative emotional episodes (Gottman et al., 2002).

### Interplay of Positive Emotion and Negative Emotion in Intentional Change

Recurrent arousal of positive emotion (activated by vision/the PEA) and negative emotion (activated by the Real Self/NEA) is a central feature of intentional change. Desired change is more lasting and effective when vision/the PEA serve as the primary focus of the change effort and when the Real Self/NEA is the secondary focus (Boyatzis, 2006; Howard, 2006). Change efforts primarily framed by arousal of vision/the PEA foster more robust learning and development than change efforts predominately framed by Real Self/NEA arousal. Promoting change through vision/PEA arousal grounds the change process in constructive cognitive and physiological processes that enhance motivation, effort, creative thinking, optimism, flexibility, resilience, and recovery from stressful thoughts, feelings, or experiences. Conversely, change promoted through NEA arousal activates defensive emotional processes concerned with minimization or prevention of losses, self-protection, and use of vigilance means (Brockner and Higgins, 2001; Higgins et al., 2001).

Grounding the change process primarily in vision/the PEA, and secondarily in the Real Self/NEA, does not compromise an actor’s engagement in capacity-building coping responses moved by the NEA. For example, a study by Isen and Reeve (2005) found that positive affect fostered intrinsic motivation without compromising involvement in meeting extrinsic requirements. When NEA arousal occurs within a change event that is framed by early PEA arousal, the individual is more resilient and flexible in overcoming challenges and stressors identified through negative emotional processing. Grounding a change process primarily in the PEA promotes the kind of change recommended by DiClemente (1999, p. 211), i.e., change that responds to environmental demands, yet is “reinforced by incentives that are owned by the individual so that they become integrated into the life of that individual.” Coaching primarily to vision/the PEA, and secondarily to the Real Self/NEA, leverages the advantages of both positive *and* negative emotion.

### Timing

Intentional change theory (Boyatzis, 2006) places great emphasis on the *timing and sequence* of affect induction. The ICT model is a non-linear process model wherein desired sustainable change is enhanced by deliberate early induction of positive affect (PEA arousal) followed by recurrent engagement in both negative emotional processing and positive emotional processing (the interplay of positive emotion and negative emotion). Early PEA arousal, followed by the interplay of positive and negative affect, organizes emotional self-regulation that enables coachees (a) to initially ground the change effort in intrinsic motivation triggered by PEA arousal; (b) as the change process unfolds, to handle salient challenges and stressors through proactive coping responses (Aspinwall and Taylor, 1997) triggered by NEA arousal; and (c) to continually re-center and reenergize through adaptive behaviors moved by PEA arousal. The unfolding PEA–NEA interplay is dynamic (self-organizing, emergent, unpredictable), iterative (repeated in fits and starts), and non-linear (multidirectional and fluctuating; it is ordered by episodic disruption, modification, and trial-and-error rather than straight linear progression).

According to the ICT perspective, a key advantage of recurrent PEA–NEA arousal is that it enables people to leverage vision and strengths (promotion-focused activity/the Ideal Self) during the change process while also dealing with problems and fears (prevention-focused activity/the Real Self). When the interplay of positive and negative emotions occurs within a change process framed by early PEA arousal, individuals access the broad range of adaptive coping behaviors moved by both kinds of affective processing. Application of vision-focused coaching (anchoring intentional change primarily in the PEA and secondarily in the NEA) is enormously helpful in situations characterized by high challenge, rapid change, or chronic stress (e.g., fierce competition, extreme financial strain, chronic illness, job loss, organizational restructuring, etc.). Again, recurrent PEA–NEA activation leverages hope, optimism, resilience, strength, and other proactive responses, *and also promotes* assessment of problems or threat, pragmatic reasoning, and self-protective coping.

Intentional change theory views on recurrent PEA–NEA arousal are undergirded in part by selected cognitive emotion research on temporal effects in emotional processing during work on behavior change. For example, Fredrickson’s (2000b, pp. 595–603) review of empirical research on the ‘peak-and-end rule’ suggests that under-researched timing effects play a significant role in determining specific ways in which positive and negative emotions influence evaluation of change requirements and future possibilities. Similarly, Gross (2001) found that specific emotion regulation strategies have different impacts depending on *when* they are employed. His research suggests that *antecedent-focused strategies* (feelings, behaviors, and physiological responses experienced early in the process of assessing a stimulus event) have more calming behavioral and physiological effects as compared to *response-focused strategies* (feelings, behaviors, and physiological responses experienced after a person’s event response-tendencies are activated). Other regulatory focus theories have demonstrated that framing a task as promotion-focused (e.g., vision/PEA priming) versus prevention-focused (e.g., improvement needs/NEA priming) triggers timing effects. In one set of studies framing a task as prevention-focused fostered preferences to initiate action earlier than did framing the task as promotion-focused (Freitas et al., 2002). Based on this result, Freitas et al. (2002) reasoned that initially framing a new activity as promotion-focused *(conceptualized in ICT as a primary focus on vision/the Ideal Self)* fosters a willingness to adopt it, but once the activity has begun, reframing it as prevention-focused *(conceptualized in ICT as a secondary focus on improvement needs/the Real Self)* fosters interest in meeting or completing the activities’ requirements.

In a related stream on non-linear dynamics in human emotion and flourishing, work on positivity ratios (Gottman, 1994; Gottman et al., 2002; Losada and Heaphy, 2004; Fredrickson and Losada, 2005; Fredrickson, 2013) contributes evidence that it is possible to describe the emotional experience of human systems *(individuals, teams, groups, organizations)* in terms of the ratio of positivity to negativity (P/N) identified through the coding of expressed emotion in spoken and verbal communication, and that high positivity ratios are associated with effective behavior, performance, and flourishing – while low positivity ratios are associated with less optimal outcomes.

While not conclusive, taken together these researches suggest that the timing and sequence of affect induction may play an influential role in the change process such that early arousal of vision/the PEA increases intrinsic interest/openness to change, and recurrent PEA–NEA arousal promotes robust work on both vision/the Ideal Self *and* improvement needs/the Real Self. Few studies have empirically tested ICT propositions on how positive and negative emotional interplay shapes the experience of coaching recipients during real-time coaching sessions, or the influence of coaches’ timing and framing *(coaching to vision/the PEA versus coaching to improvement needs/the NEA)* on the coaching process and experience. As described in the following sections, the present study contributes preliminary findings on these dynamics.

## Materials and Methods

### Design

This study examined the emotional experience of eighteen coachees during a onetime, hour-long, one-on-one executive coaching session conducted by an executive coach. Participants were randomly assigned to one of two coaching conditions: the vision/PEA condition or improvement needs/NEA condition. In the vision/PEA condition the coach used the participant’s own hopes, strengths, and desired future (Ideal Self) as the primary framework for work done in the coaching session. In the improvement needs/NEA condition the coach used the participant’s perceived improvement needs, weaknesses, and current reality (Real Self) as the primary framework. Two main hypotheses were tested. Hypothesis_1_ predicted that participants in the vision/PEA condition would show higher levels of expressed positive emotion during appraisal of 360° feedback results and discussion of change goals than recipients in the improvement needs/NEA condition. Hypothesis_2_ predicted that vision/PEA participants would show lower levels of stress immediately after the coaching session than improvement needs/NEA participants.

A secondary aim of the study was collection of self-report data on coachees’ current mood and satisfaction with the coaching session. Two secondary hypotheses were tested. Participants in the PEA condition were predicted to show higher levels of current mood and satisfaction with the coaching experience and relationship than participants in the NEA condition.

All study coaching sessions featured receipt and analysis of coachees’ 360° feedback results on the Emotional Competence Inventory (Boyatzis and Sala, 2004), a self-administered survey completed by both self and other raters. The Emotional Competence Inventory (ECI-U) measures twelve emotional intelligence competencies and two cognitive abilities linked to superior leadership and performance in the workplace. In addition to help on interpreting their 360° feedback results, all coachees received support from the coach on exploring 2–3 possible change goals. Discussion of change goals built on participants’ analysis of their feedback results. Participants’ ECI-U feedback data were not collected for research purposes; these data were used solely by the coachee and coach as a feedback resource and discussion topic during the coaching session.

Two-tailed independent *t*-tests were conducted to establish that the PEA and NEA groups were comparable with regard to population parameters (demographic characteristics, length of coaching transcripts, and ECI-U feedback results). All tests were measured at the.05 level of significance. No differences in population parameters were found between the two conditions.

Two highly experienced coaches served as coaches in this study; both coaches had extensive backgrounds in organizational management and executive coaching. Each coach conducted both PEA and NEA coaching sessions based on participants’ random assignment to the PEA or NEA coaching condition:

• To move discussion in the PEA condition, the coaches (1) framed the coaching process around the coachee’s *vision/Ideal Self* by exploring his or her future vision and perception of Ideal Self (e.g., his or her own hopes, strengths, dreams, desired future) at the beginning of the session; (2) supported the coachee during his or her assessment 360-degree feedback results and identification of strengths and weaknesses (e.g., listened to the coachee’s reactions, answered the coachee’s questions, responded to the coachee’s observations and/or requests for the coach’s feedback); and (3) helped the coachee to think about possible change goals to work on in future.
• Alternatively, in the NEA condition the coaches framed the coaching process around the coachee’s *current reality/Real Self* by exploring his or her present-day interests and perception of Real Self (e.g., his or her own current concerns, day-to-day reality, improvement needs) at the beginning of the session; (2) supported the coachee during his or her assessment of 360° feedback results and identification of strengths and weaknesses (e.g., listened to the coachee’s reactions, answered the coachee’s questions, responded to the coachee’s observations and/or requests for the coach’s feedback); and (3) helped the coachee to think about possible change goals to work on in future.

A manipulation check was conducted to establish that all PEA coaching sessions followed the PEA coaching protocol and all NEA sessions followed the NEA coaching protocol. Four raters read all coaching session transcripts in their entirety and rated each session either as in the PEA coaching condition, or in the NEA coaching condition; manipulation check transcripts included all discussion between each coach and coachee. The manipulation check indicated that inter-rater agreement was high. All raters showed acceptable and statistically significant reliability: all raters’ *r* values were above 0.7 and significant at the.001 level (two-tailed); mean agreement was 86% (0.863), median of 0.892, within a range of 0.714–1.0.

### Sample

Participants for this study were local area alumni of a Midwest U.S. dental school. Nineteen mid and late-career practicing dentists participated in the study. ^1^ Participation in the study was voluntary. The mean age of this sample was 55 years (SD = 8.7) ^2^; nearly half the participants were born between 1932 and 1949 (47.4%) and a slightly larger number between 1951 and 1967 (52.6%). Two participants were female (10%) and 17 were male (90%). The ethnic composition of the sample was 100% Caucasian; 31.6% of the participants headed group practices and 68.4% headed solo practices. All participants (100%) headed viable dental practices and were first time participants in an executive coaching assessment.

### Procedures

This study was conducted in three time stages detailed below.

#### Time 1

Random assignment to two coaching conditions, the vision/PEA condition and improvement needs/NEA condition. Administration of a pre-coaching research survey with questions on demographic characteristics and three repeated-measure self-report scales on current mood. All participants additionally competed the university version of the ECI-U, a self-administered 360° executive assessment survey.

#### Time 2

Participation in an hour-long, audio taped coaching session conducted by a confederate coach. Immediately before the start of each coaching session a self-administered, pre-coaching saliva sample was collected from the participant by the researcher. The salivary cortisol collection was conducted in a private room several doors down from the coaching office. After collection of the pre-coaching cortisol sample, the participant was taken to the coaching room and introduced to the executive coach. The coach then conducted and audio taped an hour long, one-on-one coaching session. Immediately after the coaching session the participant returned to the private room to (1) self-administer a post-coaching saliva sample and (2) complete a post-coaching survey with the three repeated-measures on current mood and a repeated measure on satisfaction with the coaching experience and relationship.

#### Time 3

One month after the coaching session, administration of the repeated-measures surveys on current mood and satisfaction with the coaching experience and relationship.

### Variables

The independent variables in this study were the coaching condition (vision/PEA condition versus improvement needs/NEA condition) and time. The time series levels within the 60-min coaching session included: 1) opening discussion on coachee’s Ideal Self (or Real Self) and overview of ECI-U Report (Segment A/*beginning 15–20 min of the 60-min coaching hour*); (2) discussion/review of coachee’s 360° feedback results (Segment B/*middle 15–20 min of the 60-min coaching hour*); and (3) closing discussion on coachee’s assessment results and possible change goals (Segment C/*ending 15–20 min of the 60-min coaching hour*). The time series levels across the overall study period included: time 1 of the study administration *(at least 1 week prior to the coaching session)*; Time 2 of the study administration *(immediately before and after the coaching session)*; and Time 3 of the study administration *(1 month after the coaching session)*. The research variables, measures, and instruments are presented in **Table 1**.

**Table 1:** Variables, measures, and instruments.

Variable	Level or measure	Instrument
**Independent variable 1:** Coaching Condition —Random Assignment	Two Levels: • PEA Condition (Ideal Self was the focus of the coaching session) • NEA Condition (Real Self was the focus of the coaching session)

**Independent Variable 2:** Time — Time Series Analysis • Three Time Sequences within the 60 Min Coaching Session • Three Time Sequences Across the Overall Study Period	Three Levels: • Beginning, middle, and ending segments of the coaching hour (segments A, B, and C of the coaching session) • TIME 1 (at least 1 week before coaching session); TIME 2 (immediately after coaching session); TIME 3 (1 month later)	

**Dependent Variable 1:** Coachee’s Level of Positive Emotion during appraisal of 360-degree feedback results and discussion of change goals.	Percentage of positive versus negative emotion words spoken by coachee during appraisal of feedback and discussion of possible change goals (during the coaching session/TIME 2).	Assessed using LIWC2001 software (Pennebaker and Graybeal, 2001).

**Dependent Variable 2:** Coachee’s Level of Stress immediately after the coaching session.	Mean change (post-pre) in level of free salivary cortisol found in the coachee’s pre-post saliva samples (TIME 2).	Clinical Laboratory Assessment.

**Dependent Variables on Current Mood (secondary measures):** Coachee’s Current Arousal State, Current Goal Directed Thinking, Current Optimism.	Coachee’s self-report on transitory arousal state, goal-directed thinking and optimism. A repeated measure administered at least 1 week before the coaching session/TIME 1; immediately after the coaching session/TIME 2; and 1 month later/TIME 3.	Assessed using the **AD ACL** (short term time instructions: *please use the rating scale next to each word to describe your feelings at this moment*); the **PANAS X** (short term time instructions: *indicate to what extent you feel this way right now, that is, at the present moment*); and the **Adult Hope Scale** (assesses goal directed thinking at a moment in time: *focus on yourself and your life at this moment. Once you have this “here and now” set, go ahead and answer each item according to the following scale*).

**Dependent Variable on Satisfaction with the Coaching Experience and Relationship (secondary measure)**	Coachee’s self-report on satisfaction with the coaching experience and relationship. A repeated measure administered in TIME 2 and 3.	Assessed using a Coaching Satisfaction Scale developed by the researcher.

**SES Variable**	Coachee’s self-report on demographic information (TIME 1).	Assessed using a self-report scale developed by the researcher.

The two major dependent variables were (1) level of positive emotion (versus negative emotion) during appraisal of 360° feedback results and discussion of change goals, and (2) level of stress immediately after the coaching session. A socioeconomic status (SES) measure was also administered. Current mood and satisfaction with the coaching experience and relationship were treated as secondary dependent variables.

### Analyses

#### Coached Person’s Level of Positive Emotion during Appraisal of 360° Feedback and Discussion of Change Goals (DV_1_)

The presence of emotion words in written and spoken speech is an indicator of cognitive emotional processing (Berry et al., 1997; Pennebaker et al., 2003). To assess positive emotion experienced by the coachee during the coaching session, all coaching sessions were audio taped and transcribed. Positive emotion experienced by the coachee was analyzed by measuring the percentage of positive versus negative emotion words in transcripts of coaching recipients’ speech during the coaching sessions. Only transcripts of the coachee’s speech were assessed for purposes of this study; the coach’s speech was not analyzed. The transcripts were content analyzed using Linguistic Inquiry and Word Count (LIWC) software (Pennebaker et al., 2003) that assesses the emotional, cognitive, structural, and process components present in verbal and written speech. Based on the research hypotheses and supporting literature, this study primarily focused on one dimension of the LIWC dictionary for analysis of participants’ coaching transcriptions: the *Affective or Emotional Processes* dimension. This dimension includes (1) positive emotions; (2) positive feelings; (3) optimism and energy; (4) negative emotions; (5) anxiety or fear; (6) anger; and (7) sadness or depression. In addition, the *Time* and *Leisure Activity* dimensions were employed. Time and Leisure dimensions are reported because they tap linguistic markers of psychological change (increase) in cognitive-analytic processing, and are cognitive processing dimensions that reached significance in the LIWC analysis. **Table 2** presents these three LIWC dimensions (Pennebaker et al., 2003, pp. 18–19).

**Table 2:** Linguistic Inquiry and Word Count (LIWC) dimensions and categories employed in the present study.

Affective or emotional processes	Abbrev (affect)	Examples (happy, ugly, bitter)	# Words 615
Positive emotions	Posemo	happy, pretty, good	261
Positive feelings	Posfeel	happy, joy, love	43
Optimism and energy	Optim	certainty, pride, win	69
Negative emotions	Negmo	hate, worthless, enemy	345
Anxiety or fear	Anx	nervous, afraid, tense	62
Anger	Anger	hate, kill, pissed	121
Sadness or depression	Sad	grief, cry, sad	72

**Time**	**Abbrev (time)**	**Examples (hour, day, clock)**	**# Words 113**

Past tense verb	Past	walked, were, had	144
Present tense verb	Present	walk, is, be	256
Future tense verb	Future	will, might, shall	14

**Leisure activity**	**Abbrev (leisure)**	**Examples (house, TV, music)**	**# Words 113**

Home	Home	house, kitchen, lawn	26
Sports	Sports	football, game, play	28
Television and movies	TV	TV, sitcom, cinema	19
Music	Music	tunes, song, CD	31

In order to establish that transcriptions of coachees’ speech in the PEA and NEA groups were comparable in length, a two-tailed independent *t*-test was conducted to test for differences between the two coaching conditions (PEA versus NEA) in mean number of pages in participants’ transcripts. No differences were detected between the length of participants’ transcripts in the PEA condition and the length of participants’ transcripts in the NEA condition [*t*(16) = 0.659, *p* = 0.52].

The objective of the LIWC analysis was to assess the percentage of positive emotion words (versus negative emotion words) present in the coached person’s speech during appraisal of 360° feedback results and discussion of change goals (DV_1_), and to collect time series data on participants’ positive versus negative cognitive emotional processing over the coaching hour (i.e., the timing and sequence of experienced positive and negative affect). The data analysis strategy was to divide the transcript of each coached person’s spoken output during the coaching session into three equal segments that reflected three basic stages in every participants’ coaching session: (1) opening discussion on the coachee’s Ideal Self (or Real Self) and an overview of the ECI-U format (first segment of the transcript/Segment A); (2) discussion of the ECI-U results, including the coachee’s initial response to his or her 360° feedback data and exploration of change goals (middle segment of the transcript/Segment B), and (3) summary discussion on the assessment results and possible change goals (last segment of the transcript/Segment C).

Linguistic Inquiry and Word Count results were analyzed using two-way mixed ANOVAs, with the independent variable of coaching condition treated as a between subjects variable (PEA, NEA) and the independent variable of time treated as a within subjects variable (transcript Segments A, B, and C). *Post hoc* tests (Mann–Whitney, Tukey) were also conducted. All tests were measured at the 0.05 level of significance. **Table 3** presents the three coaching transcript segments, and the focus of discussion in each segment.

**Table 3:** Focus of discussion during threehree transcript segments analyzed by LIWC

Segment A: opening discussion	Segment B: assessment discussion	Segment C: closing discussion/Summary
• Coachee discusses his or her personal vision and desired future (vision/PEA condition), or present reality and improvement needs (improvement needs/NEA condition), in response to the coach’s greeting and opening comments.	• Coachee evaluates mostly strengths (and a little time on weaknesses) suggested by the feedback results (vision/PEA condition), or improvement needs/opportunities suggested by the feedback results (improvement needs/NEA condition), in response to the coach’s prompts.	• Coachee continues the search for meaning in the feedback results and the exploration of future goals/action steps. • Coachee raises unaddressed questions; moves toward integration of what he or she has learned.
• Coachee asks questions and/or shares comments about the process, design, or format of the ECI-U in response to the coach’s overview of the assessment and expert guidance on how to read the ECI-U feedback report.	• Coachee also may talk about possible change goals. • Coachee invites input or feedback from the executive coach by asking the coach questions, responding to the coach’s comments, offering more thoughts/information.	• Coachee shares closing thoughts or questions in response 2o the coach’s summary of the coaching conversation.

#### Coached Person’s Level of Stress Immediately After the Coaching Session (DV_2_)

Free salivary cortisol is a biomarker of stress (Lau and Morse, 2003; Dickerson and Kemeny, 2004; Hjortskov et al., 2004; Kurina et al., 2004). To assess level of stress, a pre-coaching saliva sample was self-administered by each participant immediately before the coaching session, and a post-coaching saliva sample was self-administered immediately after the session. Saliva samples were self-administered using a standard non-invasive process for collection of free cortisol in whole saliva (Dabbs, 1991; Good et al., 2004) — i.e., by using a dental swab placed under the tongue for a timed, 2-min period (and/or until the swab has been saturated with saliva). The pre and post-coaching saliva samples were collected by this researcher and taken to a Midwest University Clinical Research Center Laboratory for analysis of cortisol levels. Change in mean cortisol levels (post–pre) was compared between the PEA and NEA groups using a two-tailed independent *t*-test (measured at the 0.05 level of significance).

#### Self-report Measures on Current Mood and Satisfaction with the Coaching Experience and Relationship (Secondary Measures)

Three self-report surveys were administered on current mood. *Current arousal state* was measured by The Activation-deactivation Adjective Checklist (AD ACL) Long Form (Thayer, 1986). *Current optimism* was measured by the Positive and Negative Affect Schedule (PANAS; Watson et al., 1988; Watson and Clark, 1994). S*atisfaction with the coaching experience and relationship* was measured using a self-report scale on coaching satisfaction developed by this researcher. Results from all self-report scales were analyzed using mixed ANOVAs, with coaching condition (NEA, PEA) and time (TIME 1, 2, and 3) as independent variables. All tests were computed using α = 0.05.

## Results

### Main Hypotheses

#### Hypothesis_1_: Level of Positive versus Negative Emotion during Appraisal of 360° Feedback

Hypothesis_1_ predicted that participants in the PEA condition would show higher levels of positive emotion during the coaching session than would participants in the NEA condition. Tests of H_1_ assessed differences between the PEA and NEA groups on three measures of positive emotion (positive emotions; positive feelings; optimism and energy) and four measures of negative emotion (negative emotions; anxiety or fear; anger; sadness or depression). Tests of H_1_ also tracked differences between the PEA and NEA groups in expressed positive versus negative emotion over three different time intervals, i.e., time series data on *changes* in positive and negative emotion during the coaching hour: *the beginning segment of the coaching session* (opening discussion on the Ideal Self/PEA or Real Self/NEA; ECI-U overview); *the middle segment of the coaching session* (discussion on 360° feedback results and possible change goals); *and the ending segment of the coaching session* (closing discussion on feedback results; integration of what was learned; summary of coaching conversation).

#### Significant Main Effects for Negative Emotions and Anger

A significant main effect was obtained for negative emotions, *F*(1,48) = 4.114, *p* = 0.048, indicating that during the coaching session the NEA group exhibited significantly higher use of words coded for negative emotion (*M* = 0.915) than did the PEA group (*M* = 0.704). *Post hoc* tests (Mann–Whitney, Tukey) were conducted in order to perform more stringent tests on Hypothesis_1_. The result of a Mann-Whitney test on the main effect for negative emotions offered suggestive evidence of a difference between the two groups, *U*(52) = 262.500, *z* = -1.698, *p* = 0.090, indicating a need for replication of the study on a larger sample size. A significant main effect also was obtained for anger, *F*(1,48) = 5.445, *p* = 0.024, indicating that the NEA group additionally exhibited significantly higher use of words coded for anger (*M* = 0.267) as compared to the PEA group (*M* = 0.133). A Mann–Whitney test on the main effect for anger was not significant, *U*(52) = 273.500, *z* = -1.520, *n.s.*

Taken together, the significant main effects for negative emotions and anger lend partial support to H_1._ Although no significant main effects were obtained for measures of positive emotion, participants in the PEA condition showed significantly lower levels of negative emotion during appraisal of 360° feedback results and discussion of change goals than participants in the NEA condition.

#### Significant Main Effect for Leisure Activity

Tests of Hypothesis_1_ yielded a main effect for leisure activity. Although not an emotional process, the result for leisure activity is reported because it reached significance in the LIWC analysis. The main effect for leisure activity, *F*(1,48) = 6.498, *p* = 0.014, indicated that the PEA group showed significantly higher use of words coded for leisure activity (*M* = 0.647) than did the NEA group (*M* = 0.355). A Mann–Whitney test on the main effect for leisure activity was significant, *U*(52) = 218.500, *z* = -2.464, *p* = 0.014.

The main effect for leisure activity offers evidence that framing the coaching session around the PEA led participants in the PEA condition to focus more on personal interests or passions they were drawn to and/or enjoyed as compared to NEA participants, whereas framing the session around the NEA resulted in the NEA group’s significantly lower attention to personal interests and passions.

#### Non-Significant Main Effects for Three Measures of Positive and Two Measures of Negative Emotion

Non-significant main effects were obtained for all three measures of positive emotion (positive emotions; positive feelings; optimism, and energy) and two remaining (out of four) measures of negative emotion (anxiety or fear; sadness or depression). **Table 4** presents all significant and non-significant main effects for tests of Hypothesis_1_.

**Table 4:** Tests of Hypothesis_1_: main effects (18 subjects; two conditions; 48 between subjects measures; significant main effects presented in shaded text).

Effect	*df*	Error Term	*F*	Significance	*M* (PEA)	*M* (NEA)
Negative emotions	1	48	4.114	0.048	0.704	0.915
Anger	1	48	5.445	0.024	0.113	0.267
Positive emotions	1	48	0.001	0.972	2.896	2.886
Positive feelings	1	48	1.032	0.315	0.521	0.636
Optimism and energy	1	48	0.057	0.812	0.475	0.457
Anxiety or fear	1	48	0.063	0.802	0.143	0.133
Sadness or depression	1	48	0.001	0.741	0.103	0.112
Leisure activity	1	48	6.499	0.014	0.647	0.355

*Computed at the 0.05 level of significance*.

#### Interaction Effects Obtained for Hypothesis_1_

Although no main hypotheses were presented on time effects, time was an independent variable in this study. Interaction effects were obtained from tests of H_1_ which provided time series data on *changes* in expressed emotion during the coaching hour, i.e., changes that took place from the *beginning segment* of the coaching session (opening discussion on the Ideal Self/PEA or Real Self/NEA; ECI-U overview), to the *middle segment* (discussion on 360° feedback results and possible change goals), to the *ending segment* (closing discussion on feedback results; integration of what was learned; summary of coaching conversation). Two significant interaction effects were found, one for sadness or depression and the other for future.

#### Interaction Effect Obtained for Sadness and Depression

**FIGURE 2:**
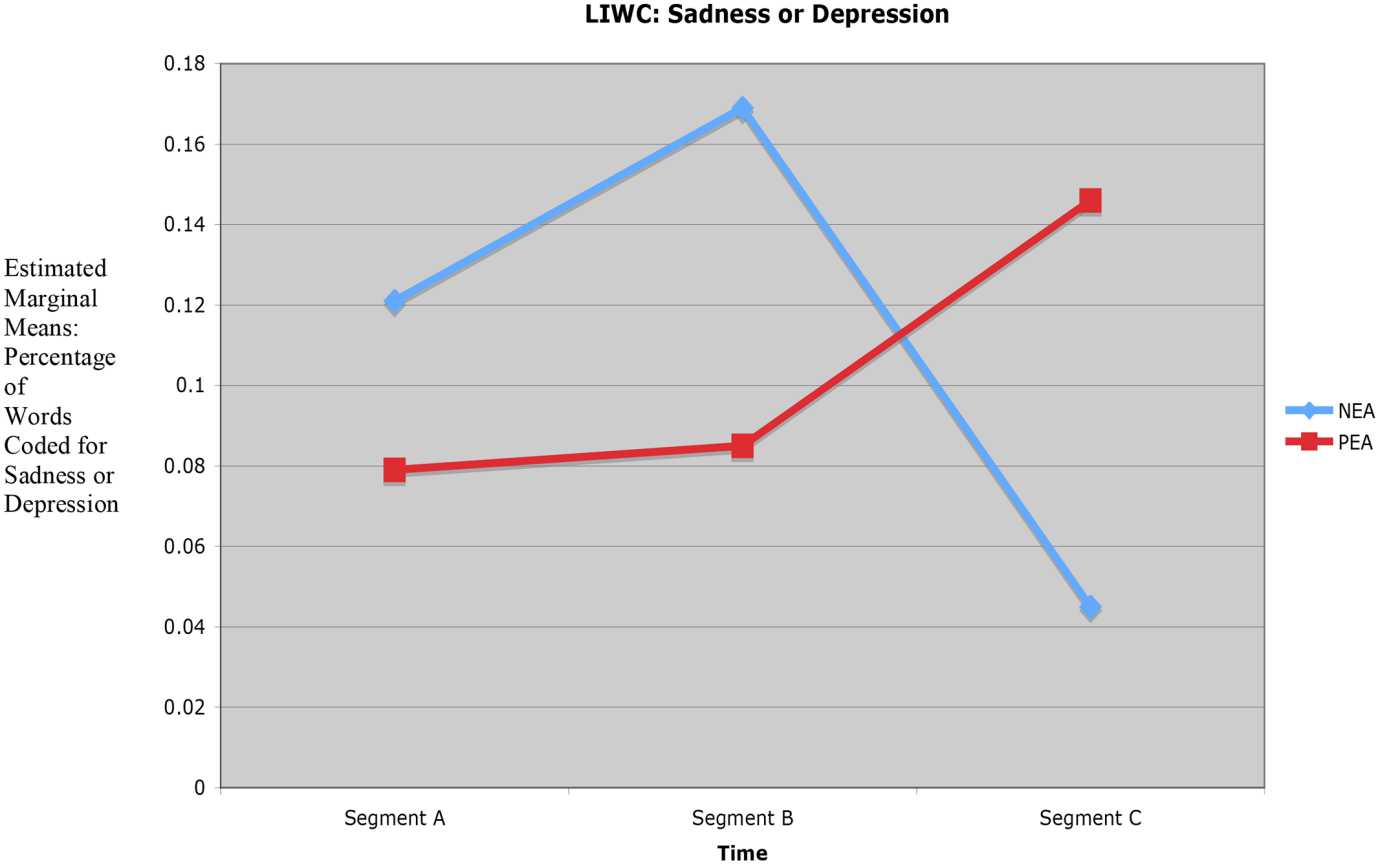
**Interaction effect for sadness or depression (18 participants, two conditions, 48 measures)**.

The significant interaction effect for sadness or depression, *F*(2,48) = 4.98, *p* = 0.011, documented NEA–PEA differences in segment-to-segment change in level of words coded for sadness or depression. In segment A of the coaching session (opening discussion on the Ideal or Real Self and overview of the ECI-U format) the NEA group exhibited a baseline level of words coded for sadness or depression (*M* = 0.121). In segment B (discussion of 360° feedback and possible change goals) the NEA group showed an even higher level of words coded for sadness or depression (*M* = 0.169). However, in segment C (closing discussion on feedback results, integration of what was learned and summary of the coaching conversation) the NEA group showed a drop in level of words coded for sadness or depression (*M* = 0.045). A different pattern of time series change was seen in the PEA group. In segment A the PEA group’s baseline level of words coded for sadness or depression was *M* = 0.079. In segment B the PEA group showed an increase in words coded for sadness or depression (*M* = 0.085), and in segment C the PEA group was even higher in words coded for sadness or depression (*M* = 0.146). *Post hoc* Tukey’s HSD tests (at *p* < 05) conducted on both interaction effects were not significant. Time series results on sadness or depression for segments A and B are in the expected direction (mean for NEA > PEA), with a reversal in segment C (mean for NEA < PEA) in both conditions during discussion of change goals. **Figure 2** presents the significant interaction effect for sadness or depression.

Although time series results on sadness or depression for segments A and B are in the predicted direction (mean for NEA > PEA), the reversal in segment C is counterintuitive (mean for NEA < PEA). One explanation for the NEA group’s sudden decline in level of words coded for sadness or depression during segment C may be that participants in the NEA group focused primarily on current reality and improvement needs throughout the coaching session, engaged in more negative emotional processing than did participants in the PEA group, hence were emotionally lifted when the session moved toward closure. Conversely, participants in the PEA group focused primarily on future possibilities and strengths, engaged in a lesser amount of negative emotional processing than did the NEA group (and perhaps were more energized by the coaching conversation than participants in the NEA group), hence were sadder to see the session come to an end. Support for this explanation is offered by the significant interaction effect on future reported next.

#### Interaction Effect Obtained for Future

**FIGURE 3:**
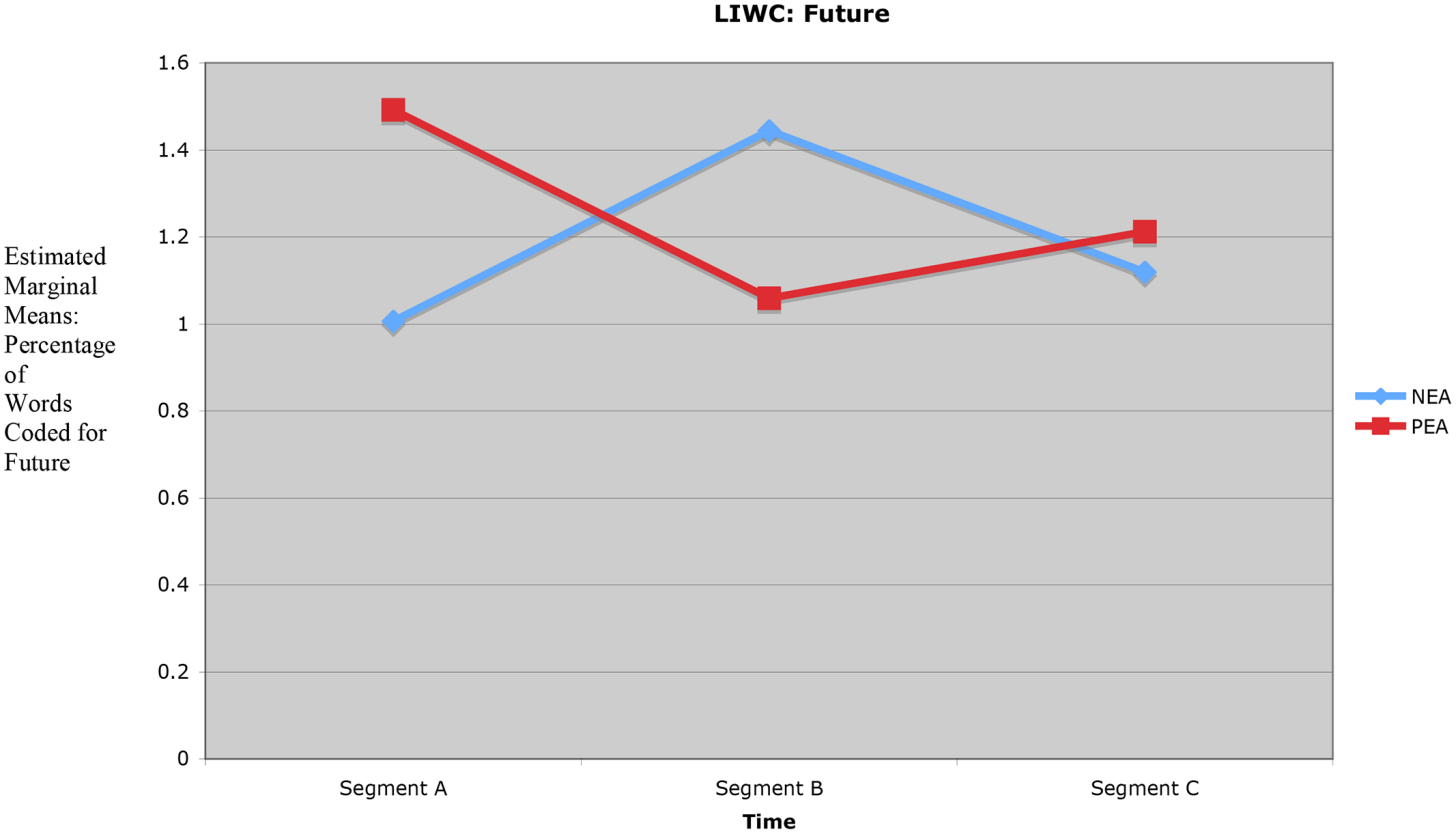
**Interaction effect for future (18 participants, two conditions, 48 measures)**.

The significant interaction effect for future, *F*(2,48) = 3.559, *p* = 0.036, documents segment-to-segment change in percentage of words coded for future. In segment A (opening discussion on the Ideal or Real Self and overview of the ECI-U format) the PEA group exhibited a baseline use of words coded for future (*M* = 1.492). In segment B (discussion of 360° feedback and possible change goals) the PEA group showed a relative decrease in use of words coded for future (*M* = 1.059). In segment C (closing discussion on feedback results, integration of what was learned, and summary of the coaching conversation) the PEA group showed a relative increase in words coded for future (*M* = 1.212). The opposite pattern was seen in the NEA group. In segment A the NEA group’s baseline use of words coded for future was *M* = 1.006. In segment B the NEA group exhibited relatively higher use of words coded for future (*M* = 1.444), and in segment C the NEA group showed a relative decrease in words coded for future (*M* = 1.119). **Figure 3** presents the significant interaction effect for future.

The significant interaction effect for future is interpreted as suggestive evidence on early arousal of the PEA (participants in the PEA condition) and NEA (participants in the NEA condition) during segment A (opening discussion on the Ideal or Real Self and overview of the ECI-U format) — and emergent interplay of positive and negative emotion in segments B and C. For example, at the beginning of the coaching session PEA participants were induced by their respective coaches to focus on the Ideal Self (hopes, strengths, desired future), and NEA participants were induced to focus on the Real Self (improvement needs, weaknesses, current reality). The significant interaction effect for future suggests that early PEA arousal led the PEA group to (1) focus on the future in segment A (indicated by a higher percentage of words coded for future as compared to the NEA group); (2) switch its focus to present reality in segment B (indicated by a relative decrease in percentage of words coded for future in segment B); and (3) refocus on the future in segment C (indicated by a relative increase in percentage of words coded for future in segment C). Conversely, early NEA arousal led the NEA group to (1) focus on present reality in segment A (indicated by a lower percentage of words coded for future as compared to the PEA group); (2) switch its focus to the future in segment B (indicated by a relative increase in percentage of words coded for future in segment B); and (3) refocus on present reality in segment C (indicated by a relative decrease in percentage of words coded for future in segment C).

Again, ICT (Boyatzis, 2006; Howard, 2006), supported by selected emotion regulation research (Freitas et al., 2002; Diamond and Aspinwall, 2003) and cognitive emotion research on non-linear dynamics in human flourishing/positivity ratios (Gottman et al., 2002; Losada and Heaphy, 2004; Sanford and Rowatt, 2004; Fredrickson and Losada, 2005; Fredrickson, 2013), proposes that intentional change is characterized by recurrent PEA-NEA arousal, and that (associated) interplay of positive emotion and negative emotion shapes the form and flow of intentional change. In the current study, segment-to-segment reversals documented by the significant interaction effects for sadness or depression and future can be viewed as suggestive evidence on recurrent PEA–NEA–PEA arousal (PEA condition) and recurrent NEA–PEA–NEA arousal (NEA condition), and related PA–NA interplay during the coaching hour.

As discussed earlier (see Role of the Positive Emotional Attractor (PEA) and Negative Emotional Attractor (NEA) in Intentional Change to Timing), negative emotions facilitate identification of situational requirements, weaknesses, and problems — and mobilize extrinsic motivation and self-protective coping. Conversely, positive emotions facilitate identification of the desired future, strengths, and personal passions — and mobilize intrinsic motivation and broad-minded coping. Overall, the PEA group’s significantly higher expression of personal interests/passions and significantly lower demonstration of negative emotions and anger, as compared to the NEA group, are interpreted as preliminary evidence that the NEA group was more narrowly focused on extrinsic requirements and self protective coping than was the PEA group, and indirect evidence that the PEA group may have experienced higher levels of positive emotion than did the NEA group. Interestingly, comparison of means for the PEA group (*M* = 2.896) versus NEA group (*M* = 2.886) on the non-significant main effect for positive emotions reveals a trend in this direction (mean for PEA > NEA.

#### Hypothesis_2_: Level of Stress Immediately After the Coaching Session

Hypothesis_2_ predicted that participants in the PEA condition would show lower levels of stress immediately following the coaching session than participants in the NEA condition. Level of stress was assessed by the measuring the cortisol levels (ug/dl) of participants in the PEA and NEA groups before and after their respective coaching sessions. Cortisol assays were analyzed by a clinical research laboratory and pre to post-coaching change in salivary cortisol was calculated for each participant (n-18). The change in mean cortisol levels (post–pre) was compared between the PEA and NEA groups using a two-tailed independent *t-*test. The *t*-test was computed using α = 0.05. No significant differences were found between the PEA group (*M* = 0.002) and NEA group (*M* = 0.036), *t*(16) = -0.508, *p* = 0.618, in level of stress immediately after the coaching session.

Although numerous studies have shown that psychological stressors can activate cortisol release (Smyth et al., 1998), research on the association between psychological stressors, affect, and salivary cortisol levels has produced inconsistent findings (Dickerson and Kemeny, 2004) on precisely when-and-how cortisol activation occurs. Given the ongoing theoretical debate on precisely what specific contexts and essential elements elicit cortisol responses, there is a need for follow-up examination of H_2_. For example, individual factors such as participants’ basal cortisol rhythms (Adam and Gunnar, 2001; Kurina et al., 2004), hypothalamic-pituitary-adrenal axis (HPA) reactivity to psychological stress (Singh et al., 1999), and responsivity to and/or mobilization for change (Brown et al., 1996) may have influenced the cortisol results reported herein. Also, socio-environmental factors such as quality of social support and social relationships have been shown to influence cortisol activation (Seeman and McEwen, 1996; Smyth et al., 1998). Consistent with these findings, it is possible that PEA and NEA participants showed no significant post–pre increase in mean cortisol levels due to receipt of (valued) social support from the executive coach in the just-concluded coaching session.

The above interpretation regarding the possible influence of social support and current mood on participants’ observed cortisol level is supported by the finding of no significant between group differences on measures of current mood (pre-coaching and post-coaching) and satisfaction with the coaching experience and relationship (immediately after the coaching session and 1 month later). These results suggest that (1) NEA participants were not more displeased with or upset by the coaching experience (i.e., digestion of 360° feedback results, consideration of change goals, help from the coach) than were PEA participants, and (2) NEA participants’ higher levels of negative emotions and anger can be viewed as evidence of emotional processing triggered by primary arousal of the coached person’s NEA/Real Self/extrinsic motivation, and secondary arousal of his or her PEA/Ideal Self/intrinsic motivation.

Similarly, the present finding may suggest that the coaching engagement simply was not a stressful experience, i.e., that the negative emotion experienced during the coaching session by participants in both groups did not reach the level of threat required to trigger a physiological stress reaction (i.e., a cascade of negative neuroendocrine activation). For example, the experience of negative emotion during a particular coaching session may not be detrimental in and of itself. Negative emotions can actually assist the coachee in feedback appraisal, recognition of problems, goal setting, and other cognitive-emotional tasks during intentional change. In this study NEA participants demonstrated a significantly higher level of negative emotions and anger than did the PEA group.

### Secondary Measures

#### Secondary Hypotheses on Current Mood and Satisfaction with the Coaching Experience and Relationship

Two secondary hypotheses were examined on current mood and satisfaction with the coaching session and relationship. Immediately after the coaching session and 1 month later, participants in the PEA condition were predicted (1) to show higher levels of positive mood and (2) to show higher levels of satisfaction with the coaching experience and relationship than were participants in the NEA condition. The study employed three self-report measures on current mood (transitory arousal state, goal directed thinking, optimism) and one self-report measure on coaching satisfaction. All tests were computed using α = 0.05. Analysis of all self-report measures yielded statistically non-significant main effects.

Non-significant results on current mood may indicate that participants were not consciously aware of small changes in momentary arousal of positive versus negative emotion during the coaching session; antecedent research has found that self-report measures and reaction tests are less effective in measuring cognitive-emotional processing than approaches that employ linguistic analysis (Pennebaker and Lay, 2002).

The non-significant result on satisfaction with the coaching session and relationship may suggest that both groups were satisfied with the coaching experience despite present findings on lower levels of negative emotion and anger in the PEA group versus NEA group. One explanation for the non-significant finding on coaching satisfaction is that study participants were mid-career professionals heading competitive dental practices. They also were first-time recipients of an executive coaching assessment. As practicing dentists and heads of group practices (31.6%) and solo practices (68.4%), participants in both conditions may have viewed the coaching session as a rare opportunity to receive executive coaching support and 360° feedback on their interpersonal abilities and dental team leadership skills.

In any case, non-significant findings on the self-report measures (current mood; satisfaction with the coaching experience and relationship) highlight the potential importance of the coach’s ability to anchor a coaching session in the coached person’s PEA, and to give secondary attention to the NEA. This ability may be critical because the coached person may or may not be aware of the restorative benefits of positive emotional processing, nor able to optimize the long term benefits of grounding in the PEA.

## Discussion

This study was the first ICT study to empirically examine the differential impact of inducing the coached person’s vision/PEA versus improvement needs/NEA during a real-time coaching session on appraisal of 360° feedback results and exploration of change goals. Findings showed that participants primarily coached to vision/PEA experienced a significantly lower level of negative emotions (*p* = 0.048) and anger (*p* = 0.024) during the coaching session as compared to participants primarily coached to improvement needs/NEA. In addition, the vision/PEA group focused significantly more on leisure activity (personal interests and passions such as competitive car racing, athletics, volunteerism, travel) than did improvement needs/NEA participants (*p* = 0.014).

Time series analysis of the beginning (segment A), middle (segment B), and ending (segment C) sequences of the coaching sessions offered suggestive evidence that both groups experienced notable changes in positive and negative emotional processing during the coaching hour. A significant interaction effect for level of sadness or depression (*p* = 0.011) documented segment-to-segment change in expressed sadness; and a significant interaction effect for future documented segment-to-segment change in expressed attention to the future (*p* = 0.036).

Consistent with social complexity perspectives on the capacity of small occurrences to have large impacts over time (Casti, 1994), the present findings are interpreted as preliminary evidence that framing a coaching session in the coached person’s vision/PEA (versus Real Self/ NEA) enhances work on intentional change. Although participants in both groups appeared to benefit from the coaching experience (no significant between group differences were found on level of stress, post-coaching current mood, or post-coaching satisfaction with the coaching experience and relationship), the data suggest that the PEA group demonstrated significantly lower levels of expressed negative emotions and anger during the coaching hour as compared to the NEA group. Given the comparatively elevated levels of negative emotion and anger exhibited by the NEA group, it is reasonable to suggest that framing a coaching session in vision/the PEA may foster a higher level of positivity (Gottman, 1994; Gottman et al., 2002), leverage the broaden-and-build benefits of positive emotion/positivity (Fredrickson, 1998, 2000a,b, 2001, 2003, 2013; Fredrickson and Losada, 2005), and create a richer emotional space (Losada and Heaphy, 2004) than was experienced by the NEA group. The significant between-group difference on discussion of personal interests or passions (i.e., the measure on leisure activity) lends support to this idea. Last, significant findings on segment-to-segment changes in sadness or depression and future (time series changes observed in both groups) are interpreted as suggestive evidence on recurrent mobilization of positive *and* negative emotion during intentional change.

### Implications for Research

A core aim of this study was to conduct an empirical investigation on specific ways in which the coached person’s cognitive affective processing influences the form and flow of his or her work on desired change during real time coaching sessions — and specific ways in which his or her coach can leverage this processing to promote sustained change. Few coaching studies have empirically tested propositions from a theoretical model on the differential influence of positive versus negative emotional processing in intentional change. Moreover, few empirical studies have explored what happens in live coaching sessions. This study contributes preliminary findings upon which to build future research on the impact of positive versus negative emotional processing in real-time coaching contexts, empirical work of potential relevance to emotion and coaching researchers alike.

Findings showed that framing a coaching session in vision/the PEA resulted in the vision/PEA group’s comparatively lesser experience of negative emotions and increased discussion of personal interest/passions as compared to the improvement needs/NEA group. On the other hand, framing the session in improvement needs/the NEA resulted in comparatively higher experience of negative emotions and lesser focus on personal interests/passions. Even so, these findings leave open the question of whether PEA framing directly activates positive emotions, as was predicted in Hypothesis_1_. Based on significant main effects and significant *post hoc* tests, the findings are viewed as suggestive evidence that vision/PEA framing does activate positive emotions and their beneficial effects, and that replication of the present study on larger sample sizes will yield the predicted results (i.e., higher levels of positive emotion in coaching sessions framed by early vision/PEA induction).

Also meriting further investigation are current findings on the beneficial effects of recurrent PEA arousal. This study found that framing the coaching session primarily in vision/the PEA, with secondary focus on the NEA, resulted in recurrent arousal of the PEA during the coaching hour, lower levels of negative emotion/anger, and elevated expression of personal interests/passions. Conversely, participants who received coaching framed primarily in improvement needs/the NEA experienced higher levels of negative emotions/anger and lesser discussion of personal interests/passions. More study is needed to establish that recurrent PEA arousal activates positive emotions as opposed to merely decreasing the level of negative emotions, and that predominant NEA arousal does not foster sufficient PEA recurrence to optimize recovery from the harmful effects of negative emotional processing.

Present findings on time series change in expressed emotion during the beginning, middle, and ending segments of study coaching sessions offered indirect evidence on the interplay of positive and negative emotions during the coaching hour. Questions remain about precisely *how* primary focus on vision/the PEA and secondary focus on improvement needs/the NEA shapes this interplay and assists behavior change. Time series research on coaching is needed to further examine the relationship between positive and negative emotional interplay in real time coaching sessions – and the optimal balance between positive versus negative emotional processing during intentional change.

Overall, for recipients of an hour-long coaching session, early vision/PEA arousal (1) fostered a significantly lower level of negative emotions and significantly greater consideration of personal passions (as compared to early improvement needs/NEA arousal); (2) led to significantly lower levels of anger during initial appraisal of feedback results (as compared to early NEA arousal); and (3) generated different patterns of time series change in emotional processing over the coaching hour (as compared to patterns generated by early NEA arousal). Taken together, these results suggest that vision/PEA arousal versus improvement needs/NEA arousal impact the coaching process in quite different ways; that the coach’s initial framing of the session predominantly in the PEA (or, alternatively, predominantly in the NEA) fosters emotional processing that is driven by this initial framing; and that both the PEA (and associated positive emotions) and NEA (and associated negative emotions) play an important and recurrent role in shaping the change process. Further study on these outcomes will enable researchers to shed more light on the differential impact of the PEA versus NEA on intentional change, and how to leverage the benefits of both emotional attractors.

### Implications for Practice

Both researchers and practitioners have called for empirical research that can ground coaching practice in tested theory and techniques. Current findings suggest that coaches can benefit from better understanding the importance of tapping intrinsic motivation and passions through coaching to vision/the PEA. Coaches additionally would benefit from better understanding how to leverage the long-term advantages, and restorative benefits, of positive emotions during coaching engagements. The findings also highlight coaches’ need to appreciate the impact of timing effects on coaching intentional change, and how coaches can play a critical role in calibrating the pace and focus of work on intentional change. Early arousal of the coachee’s PEA, accompanied by recurrent PEA–NEA induction, may help coachees be/become more creative, optimistic, and resilient during a given change process. Also, primary focus on vision/PEA and secondary focus on improvement needs/NEA may better equip coaches and coaching recipients to work together on building robust learning, development, and change.

### Limitations

Findings are based on participants’ response to a one-time, hour-long coaching session. As first-time recipients of an executive assessment, research participants may have been unfamiliar with management coaching and may not have known precisely what to expect. The population was limited to mid-career medical professionals (practicing dentists/dental practice heads) and did not include individuals from management or other professional sectors; it is not yet clear whether these findings can be generalized to other populations. Also, the study is based on 18 coaching sessions and needs to be replicated on larger samples.

## Conflict of Interest Statement

The Reviewer John Paul Stephens declares that, despite being affiliated to the same institution as the author Anita Howard, the review process was handled objectively and no conflict of interest exists. The author declares that the research was conducted in the absence of any commercial or financial relationships that could be construed as a potential conflict of interest.

## Footnotes

1 Although 19 participants were randomly assigned to study coaching conditionsand completed all research procedures, one coaching session was not recordeddue to malfunction of the tape recorder. As a result only 18 coaching participantswere included in the final data analysis.

2 Five participants did not indicate their year of birth.
